# In Vitro Anticancer Activity of Extracellular Vesicles (EVs) Secreted by Gingival Mesenchymal Stromal Cells Primed with Paclitaxel

**DOI:** 10.3390/pharmaceutics11020061

**Published:** 2019-02-01

**Authors:** Valentina Coccè, Silvia Franzè, Anna Teresa Brini, Aldo Bruno Giannì, Luisa Pascucci, Emilio Ciusani, Giulio Alessandri, Giampietro Farronato, Loredana Cavicchini, Valeria Sordi, Rita Paroni, Michele Dei Cas, Francesco Cilurzo, Augusto Pessina

**Affiliations:** 1 CRC StaMeTec, Department of Biomedical, Surgical and Dental Sciences, University of Milan, 20133 Milan, Italy; valentina.cocce@guest.unimi.it (V.C.); anna.brini@unimi.it (A.T.B.); aldo.gianni@unimi.it (A.B.G.); giampietro.farronato@unimi.it (G.F.); loredana.cavicchini@unimi.it (L.C.); 2 Department of Pharmaceutical Science, University of Milan, 20133 Milan, Italy; silvia.franze@unimi.it; 3 IRCCS Orthopedic Institute Galeazzi, 20161 Milan, Italy; 4 Maxillo-Facial and Dental Unit, Fondazione Ca’ Granda IRCCS Ospedale Maggiore Policlinico, 20122 Milan, Italy; 5 Department of Veterinary Medicine, University of Perugia, 06123 Perugia, Italy; luisa.pascucci@unipg.it; 6 Laboratory of Clinical Pathology and Medical Genetics, Fondazione IRCCS Istituto Neurologico “C. Besta”, 20133 Milan, Italy; emilio.ciusani@istituto-besta.it; 7 Cellular Neurobiology Laboratory, Department of Cerebrovascular Diseases, IRCCS Neurological Institute C. Besta, 20133 Milan, Italy; giulio.alessandri@istituto-besta.it; 8 Unit of Orthodontics and Paediatric Dentistry, Fondazione Ca’ Granda IRCCS Ospedale Maggiore Policlinico, 20122 Milan, Italy; 9 Diabetes Research Institute, IRCCS San Raffaele Scientific Institute, 20132 Milan, Italy; sordi.valeria@hsr.it; 10 Department of Health Sciences of the University of Milan, 20142 Milan, Italy; rita.paroni@unimi.it (R.P.); michele.deicas@unimi.it (M.D.C.)

**Keywords:** gingiva mesenchymal stromal cells, paclitaxel, squamous cell carcinoma, drug delivery, exosomes

## Abstract

Interdental papilla are an interesting source of mesenchymal stromal cells (GinPaMSCs), which are easy to isolate and expand in vitro. In our laboratory, GinPaMSCs were isolated, expanded, and characterized by studying their secretome before and after priming with paclitaxel (PTX). The secretome of GinPaMSCs did not affect the growth of cancer cell lines tested in vitro, whereas the secretome of GinPaMSCs primed with paclitaxel (GinPaMSCs/PTX) exerted a significant anticancer effect. GinPaMSCs were able to uptake and then release paclitaxel in amounts pharmacologically effective against cancer cells, as demonstrated in vitro by the direct activity of GinPaMSCs/PTX and their secretome against both human pancreatic carcinoma and squamous carcinoma cells. PTX was associated with extracellular vesicles (EVs) secreted by cells (EVs/PTX), suggesting that PTX is incorporated into exosomes during their biogenesis. The isolation of mesenchymal stromal cells (MSCs) from gingiva is less invasive than that from other tissues (such as bone marrow and fat), and GinPaMSCs provide an optimal substrate for drug-priming to obtain EVs/PTX having anticancer activity. This research may contribute to develop new strategies of cell-mediated drug delivery by EVs that are easy to store without losing function, and could have a superior safety profile in therapy.

## 1. Introduction

A large number of sources have been considered to isolate and expand mesenchymal stromal cells (MSCs), by taking into account the accessibility and availability of stem cells as well as their biological characteristics [1,2]. Most of these studies were conducted on MSCs derived from bone marrow, adipose tissue, umbilical cord blood, and, more recently, also from oral material [3,4,5]. Among MSCs from the oral compartment, gingival MSCs can be easily isolated, since this tissue can be removed during dental crown lengthening and periodontal surgical procedures [4,6]. As reported, MSCs from gingival papilla (GinPaMSCs) have an interesting phenotype previously described in detail [7], a relatively low osteogenic differentiation ability [8], and have significant wound healing properties that allow tissue repair without producing significant scarring [9]. Of course, most studies on MSCs have been addressed to clinical applications for regenerative medicine. The ability of MSCs to incorporate molecules and use them as a tool for drug delivery has also been proposed and intensively studied in the last years. In fact, the drug delivery mediated by MSCs can have significant advantages in comparison to the systemic administration of free chemotherapy drugs, because MSCs may migrate towards inflammatory microenvironments and accumulate in the tumor sites [10], opening up the possibility of new therapeutic anticancer strategies based on MSCs, including engineered MSCs [11,12]. Furthermore, when used in loco-regional treatments, these systems can improve the effective drug concentration in the cancer tissue and, therefore, reduce the adverse toxic effects. Moreover, vesicles might be involved in the clearance of drugs. In fact, harmful substances such as cytotoxic drugs may be associated to the vesicles, and vesicle shedding acts as a mechanism of drug expulsion. In particular, lipophilic drugs are shuttled to the plasma membrane via vesicle-mediated traffic for the final elimination [13,14]. MSCs from different sources can uptake and release drugs without any genetic manipulation, and can incorporate a significant amount of a chemotherapeutic drug (e.g., paclitaxel) that is subsequently released in quantities sufficient to affect cancer cell proliferation both in vitro and in vivo [15,16]. This capacity has also been demonstrated for mesodermal cells isolated from gingival interdental papilla [17] and dental pulp [18], suggesting that GinPaMSCs could be an interesting potential tool for cytotherapy based on cell-mediated drug delivery. Furthermore, MSC secretomes (including extracellular vesicles (EVs)) have been recently reported to be a possible alternative to MSCs for therapeutic purposes [19], being able to release accumulated drugs not only as free molecules, but also associated to exosomes and/or microvesicles [20]. Based on these observations, the present study aimed to assess the anticancer activity of secretomes from both untreated and paclitaxel (PTX)-primed GinPaMSCs, by demonstrating that both PTX-loaded GinPaMSCs and the corresponding extracellular vesicles (EVs/PTX) were active against cancer cells. This study, performed on tongue squamous cell carcinoma cell line SCC154, provides a strong proof of concept, suggesting a possible application of the procedure to collect PTX-associated EVs from drug-primed GinPaMSC working as “natural anticancer liposomes”. Among the possible different MSC sources, gingiva could be the source of choice, since MSCs obtained with minimal invasive procedures are easy to expand, with high anatomical homology to treat oral neoplasia. 

## 2. Materials and Methods 

### 2.1. Mesenchymal Stromal Cells

Human MSCs were isolated from gingival papilla, and after expansion were characterized as previously described [7,17]. Briefly, samples of gingival tissue obtained from reductive gingivoplasty were minced with surgical scissors, treated for 3 h with type I Collagenase (50 U/mL, Life Technologies, Monza, Italy) at 37 °C under stirring conditions, and the cells were centrifuged at 300× *g* for 10 min. The cells present in the pellets were cultured in a 25 cm^2^ flask in Dulbecco’s modified Eagle’s medium with high glucose (DMEM HG) + 10% foetal bovine serum (FBS) and 1% l-glutamine (Euroclone, UK), at 37 °C in atmosphere of air + 5% CO_2_. Primary cultures were then studied to evaluate the population doubling time (PDT), clonogenicity (CFU-F), and expression of the mesenchymal stem cell markers (CD73, CD90, and CD105). GinPaMSCs showed a mild expression of CD14, but they were CD45-negative and able to differentiate into osteogenic, adipogenic, and chondrogenic lineages.

### 2.2. PTX Loading in GinPaMSCs

To load GinPaMSCs with PTX, the cells were primed with a high amount of drug according to a standardized procedure previously described [7,15,17]. Briefly, cultures were obtained by seeding 2 × 10^4^ cells/cm^2^, and after 72 h, cells were exposed to 2 µg/mL PTX for 24 h. Then, after washing twice with phosphate buffered saline (PBS), the cell monolayer was trypsinized, washed in Hank’s solution (HBSS)( Euroclone, Pero, Italy), and the PTX-primed cells (GinPaMSCs/PTX) were seeded in a 25 cm^2^ flask in DMEM HG with 10% FBS and 2 mM l-glutamine (Euroclone, Pero, Italy) to release the drug. After 48 h of incubation into conditioned media (CM), PTX-loaded GinPaMSCs (GinPaMSCs/PTX/CM) were collected and tested in vitro for their anti-proliferative activity on different tumor cell lines (see Section 2.7). In particular, human pancreatic adenocarcinoma cell line CFPAC-1 was used as a standard laboratory assay according to the method reported below. CM from untreated MSCs were used as control.

### 2.3. Cell Cycle Analysis

A cell cycle study was performed by starting from GinPaMSCs after synchronization, obtained by serum starvation (48 h of culture in medium containing 0.5% FBS). Then, the cells were treated with PTX in 25 cm^2^ flasks according to the above described standard conditions. DNA content for cell cycle phase detection was estimated by comparing untreated cells, 24 h PTX-primed cells, and cells trypsinized (i.e., drug uptake-phase), washed, and subcultured in the absence of PTX for 24 h (i.e., drug releasing phase). Briefly, cells were suspended in phosphate buffered saline (PBS) and fixed with 96% (*v*/*v*) ethanol for 1 h at 4 °C. After a PBS wash, cells were suspended in propidium iodide (50 μg/mL) in PBS. Cells were incubated overnight at 4 °C and analyzed by FC (FacsVantageSE, Becton-Dikinson, Franklin Lakes, NJ, USA).

### 2.4. Secretome Analysis and Extracellular Vesicles (EVs) Collection

To collect EVs, the medium of 72 h cultures of GinPaMSCs (6 × 10^4^ cells/cm^2^; both not primed and primed with PTX, as above described) was replaced with basal medium, namely DMEM HG + 1% l-glutamine without foetal bovine serum. Then, CM of cell cultures, the secretome, were collected at 24 and 48 h of incubation at 37 °C, 5% CO_2_. To separate the PTX-loaded EVs from free PTX, aliquots of secretome were centrifuged on a 100 kDa filter device (Microsep Advance Centrifugal Devices, Life Sciences, Port Washington, NY, USA) at 5000× *g* for 15 min. The two fractions (i.e., EV: F > 100 kDa; free PTX: F < 100 kDa) were collected and characterized by the physico-chemical and biological assays reported below, using the whole secretome as control.

### 2.5. Extracellular Vesicles (EVs) Characterization

#### 2.5.1. Phospholipids

The phospholipid concentrations present in the EVs were estimated as phosphate content, using the Rouser method and sodium dihydrogen phosphate as standard [21]. Test and standard samples were inserted in separate Pyrex glass tubes and heated at 100 °C until complete evaporation. An empty tube was used as control. To liberate phosphates, samples and standards were cleaved by addition of 300 µL of 70% perchloric acid and heated at 200 °C for 20 min. Then, 1 mL of purified water and 400 µL 1.25% *w*/*v* ammonium molybdate were added to each tube and mixed vigorously. Finally, 400 µL of 5% *w*/*v* ascorbic acid (Sigma-Aldrich, Darmstadt, Germany) was added and mixed before heating at 100 °C for 5 min. In the presence of phosphate, samples turned blue and the absorbance at 820 nm was measured. The phospholipid concentration in EVs were estimated to be proportional to the absorbance of a 40 nmol/µL standard. All analyses were performed at least in triplicate.

#### 2.5.2. Particle Size and ζ-Potential

Particle size distribution and ζ-potentials of samples were determined using a Zetasizer (Nano-ZS, Malvern Instrument, Malvern, Worcestershire, UK). To perform dynamic light scattering (DLS) analyses, samples were opportunely diluted with ultrapure MilliQ^®^ water to avoid the interference due to the culture medium coloration. Particle size measurements were carried out using a disposable cuvette and a detection angle of 173°. ζ-potentials were measured in the same sample. The results are expressed as the mean and standard deviation of three measurements. 

#### 2.5.3. EVs Concentration 

The concentration of EVs was determined by a nanoparticle tracking assay (NTA, Nanosight NS300, Malvern Instrument, Malvern, Worcestershire, UK). A 25 μL sample was diluted with PBS to 1 mL and analyzed at 25 °C. The capture of the images was performed according to the following setting: Camera shutter 31.48 ms; 24.98 fps; detection threshold 7 multi. The results are expressed as the mean of five determinations.

#### 2.5.4. Transmission Electron Microscopy (TEM)

The CM were analysed by a previously described TEM procedure [22]. Briefly, 20 µL of EV suspension was placed on Parafilm. A formvar-coated copper grid (Electron Microscopy Sciences, Hatfield, PA, USA) was gently placed on the top of the drop for about 60 min in a humidified chamber. Grids were then washed in 0.1 M cacodylate buffer (CB) at pH 7.3 and finally fixed for 10 min with 2.5% glutaraldehyde (Fluka, St. Louis, MO, USA) in CB. After washing in CB, EVs were contrasted with 2% uranyl acetate. The grids were then air dried and observed under a Philips EM208 transmission electron microscope (TEM) equipped with a digital camera (University Centre for Electron Microscopy (CUME), Perugia, Italy).

### 2.6. Mass Spectrometry Analysis

PTX extraction and purification from secretomes was performed by liquid–liquid extraction (LLE). Aliquots of 500 µL of secretome, or fractions, were added with 100 µL of internal standard (0.1 µg/mL PTX-d5, Cayman Chemicals, Ann Arbor, MI, USA) and with toluene in a ratio of 1:2 (*v*/*v*). After sonication for 30 min at 40 °C, samples were vigorously shaken at 50 oscillations/s for 15 min, then centrifuged at 10,000 rpm for 2 min. The organic phase was evaporated and re-dissolved with 100 µL methanol, and 10 µL was injected for LC–MS/MS analysis. The multiple reaction monitoring (MRM) analysis was run on a HPLC Dionex 3000 UltiMate (Thermo Fisher Scientific, Waltham, MA, USA) coupled to a tandem mass spectrometer AB Sciex 3200 QTRAP (AB Sciex S.r.l., Milan, Italy). Separation was attained on a reversed-phase analytical column (Luna^®^, 3 μm, C18(2) 50 × 2 mm, Phenomenex, CA, USA), with a linear gradient between eluent A (water + 5 mM ammonium formate + 0.1% formic acid) and eluent B (acetonitrile + 0.1% formic acid). After 2 min at 20%, eluent B was increased to 95% in 4 min, held for 0.5 min, taken back to the initial conditions in 0.5 min, and kept for 2 min at 20%. The flow rate was 0.4 mL/min, and the autosampler and the column oven were kept at 15 °C and 30 °C, respectively. The representative MRM transitions were 854.5 > 286.1 (PTX) and 859.4 > 291.5 (IS, PTX-d5).

### 2.7. Tumor Cell Lines

Human pancreatic adenocarcinoma cell line CFPAC-1 [23,24], glioblastoma multiforme cell line T98G [25], human meshotelioma cell line M20 [26], and human squamous cell carcinoma line SCC154 [27] were provided by Centro Substrati Cellulari (ISZLER, Brescia, Italy). Cells were maintained in the complete medium (Iscove modified Dulbecco’s medium (IMDM) for CFPAC-1, T98G, and M20; DMEM HG for SCC154) supplemented with 10% FBS, by 1:5 weekly dilution. All reagents were provided by Euroclone, Pero, Italy.

### 2.8. In Vitro Anticancer Assays

The inhibitory effects of secretomes from drug-loaded GinPaMSCs, as well as the F > 100 kDa and F < 100 kDa fractions, were evaluated on the proliferation of different cancer cell lines by the MTT assay as previously described [15], taking the optical density (OD) of the cancer cell treated with EVs-unloaded GinPaMSCs as a control. The inhibitory concentration (IC_50_) was determined according to the Reed & Muench formula [28]. To study the interaction between CFPAC-1 and GinPaMSCs, a rosette adherence assay was performed [29,30]. Briefly, 5 × 10^5^ CFPAC-1 were mixed with GinPaMSCs or GinPaMSCs/PTX in a conical tube with 0.5 ml of IMDM + 5% foetal bovine serum (FBS; Lonza, I). The cell suspension was incubated at 37 °C in air + 5% CO_2_ without stirring. After 24 h, 20 µL suspensions were collected by a micropipette from the pellet lying on the tube bottom, and then transferred on a slide in order to evaluate the rosette formation under inverted microscope (Leitz, Germany) at 100× and 200× magnifications.

### 2.9. Statistical Analysis

Data are expressed as average ± standard deviation (SD). Differences between mean values were evaluated according to Student’s *t*-test performed by the GRAPHPADINSTAT program (GraphPad Software Inc., San Diego, CA, USA) or ANOVA, followed by the Tukey post-hoc analysis (OriginPro 2017, Origin US, Nothampton, MA, USA). *p* values ≤ 0.05 were considered statistically significant. The linearity of response and the correlation were studied using regression analysis, by Excel 2013 software.

## 3. Results

### 3.1. Sensitivity of GinPaMSCs to the Cytotoxic Activity of PTX 

**Figure 1:**
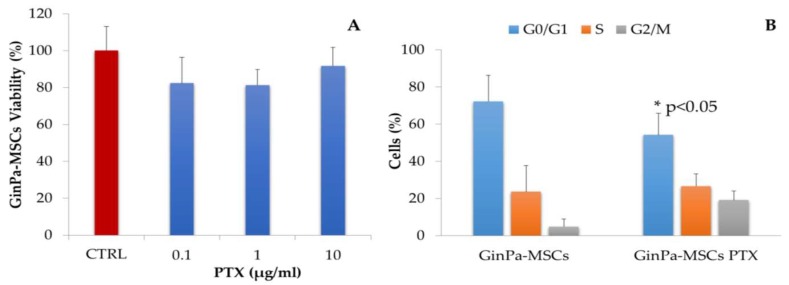
(**A**) Sensitivity of gingival papilla mesenchymal stem cells (GinPaMSCs) to cytotoxic activity of paclitaxel (PTX) was evaluated as cell viability at 24 h of treatment in the presence of three increasing logarithmic concentrations of the drug. The effect is expressed as percentage of the optical density measured in cultures that did not receive PTX (considered as 100%). The histogram shows the mean ± standard deviation (SD) of three independent experiments. (**B**) The histograms show the cell cycle-phase distributions of GinPaMSCs before and after treatment with 2000 ng/ml of PTX for 24 h (GinPaMSCs/PTX). Each value represents the mean ± standard deviation (*n* = 3).

The sensitivity of GinPaMSCs to PTX, tested by a 24 h cytotoxic MTT assay at three logarithmic dosages of 0.1–1 and 10 µg/mL (Figure 1A), confirmed the significant resistance of MSCs to the toxic effect of PTX that did not affect significantly the cell viability. The cell cycle analysis, studied on GinPaMSCs treated with a standard dosage of 2 µg/mL for 24 h (Figure 1B), indicated a decreased number of cells in phase G0, accompanied by a significant (*p* < 0.05) increase of cells in the G2/M phase. This is compatible with the known mechanism of action of PTX, which inhibits the cell proliferation in G2/M.

### 3.2. Effects Exerted on Tumor Cell Growth by Cytokines Detected in the GinPaMSCs Secretome

**Figure 2:**
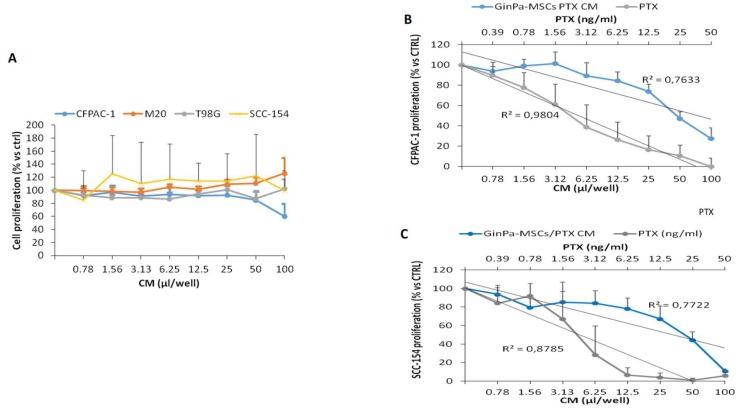
The activity of the GinPaMSc secretome was tested on the proliferation of four cancer cell lines (**A**). The GinPaMSCs/PTX secretome anticancer activity (blue line) and PTX solution (black line) against (**B**) pancreatic cancer cells CFPAC-1 and (**C**) squamous cell carcinoma SCC-154. The data represents the mean ± standard deviation of three independent experiments.

The treatment of GinPaMSCs with PTX did not significantly modify the pattern of cytokine production, except for an increase of IL8 and MIF and a decrease of SCGFb. The secretome of GinPaMSCs did not modulate the in vitro cancer cell proliferation independently of the cell line (Figure 2A). On the contrary, the secretome of cells primed with PTX (GinPaMSCs/PTX) exerted a dramatic dose–response inhibition of CFPAC-1 (pancreatic carcinoma) and SCC-154 (squamous carcinoma). Indeed, for both the cell lines, the regression analysis showed high coefficients of correlation (*R*^2^), comparable to that of PTX (Figure 2B,C).

### 3.3. Direct Anticancer Activity by GinPaMSCs/PTX

**Figure 3:**
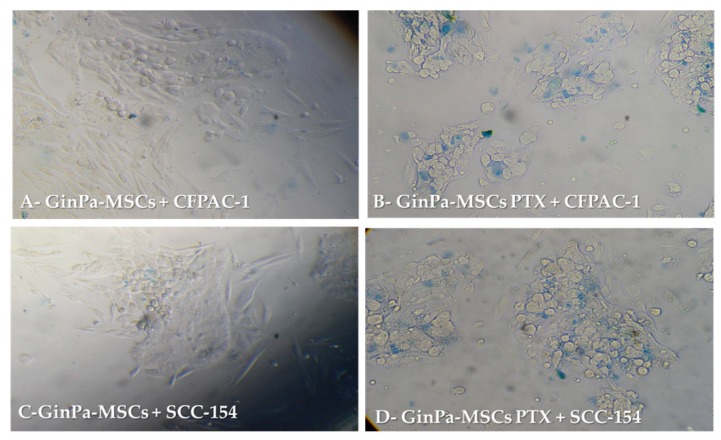
Direct anticancer activities of GinPaMSCs and GinPaMSCs/PTX secretomes against pancreatic cancer cells CFPAC-1 and squamous cell carcinoma SCC-154, evaluated by trypan blue in a MSCs–tumor cells co-culture system. (Panels A and C = 100× magnification, panels B and D = 200× magnification).

The anticancer activity against CFPAC-1 and SCC-154 was confirmed by a 24 h co-culture of MSCs and cancer cells (Figure 3). Cancer cells co-cultured with GinPaMSCs did not show any sign of toxicity (Figure 3A,C), whereas many dead cancer cells were detected in the presence of GinPaMSCs/PTX, as confirmed by intracellular trypan blue uptake (Figure 3B,D).

### 3.4. Characterization of EVs from GinPaMSCs and GinPaMSCs/PTX Secretome

**Figure 4:**
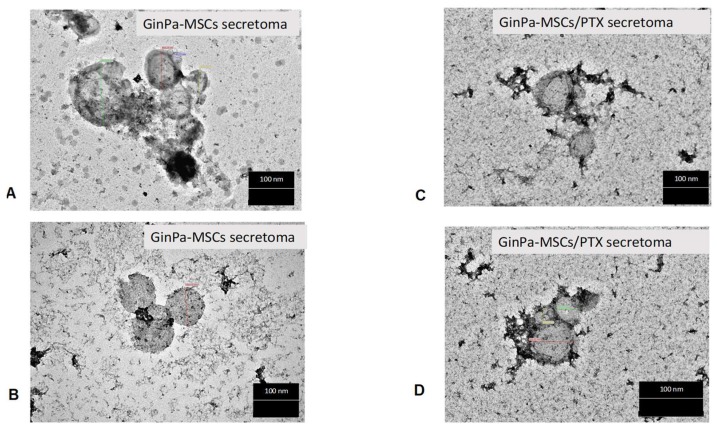
TEM analysis of extracellular vesicles (EVs) isolated from the secretome of cell cultures. No differences are evidenced in round-shaped morphologies of EVs from GinPaMSCs (**A**,**B**) and GinPaMSCs/PTX (**C**,**D**) with regard to size, shape, or electron density. Scale bar 100 nm.

TEM analysis of the GinPaMSCs secretome suggested the presence of EVs and exosomes with different sizes ranging from 50 to 500 nm. The treatment of GinPaMSCs with PTX did not modify the morphology of the EVs/exosome presence in either of the conditioned media (Figure 4).

The size distribution analysis of the isolated secretomes of GinPaMSCs was deepened both by DLS and NTA. The results of DLS analysis showed the presence of a population of microvesicles, with a particle size ranging between 200 and 300 nm, in the samples obtained from both the control and PTX-treated MSCs (Table 1). Purification by ultrafiltration led to the formation of large particles, but this increment of particle size was significant only in the case of GinPaMSCs samples (Tukey test *p* = 0.0003). This variation was not considered as a sign of particle aggregation, since the ζ-potentials showed only slight changes after purification values, maintaining the same trend (Table 1). The ζ-potential of the isolated EVs was quite negative (Table 1), and this is in line with the abundance of phosphatidylserine [31]. 

The presence of phospholipids, which do not represent the totality of the lipid composition of extracellular vesicles, was confirmed by the Rouser assay (Table 1). No trace of vesicles was instead found in the filtrate, confirming that the purification process did not lead to the loss of some secretomes, but only allowed the removal of free PTX. 

**Figure 5:**
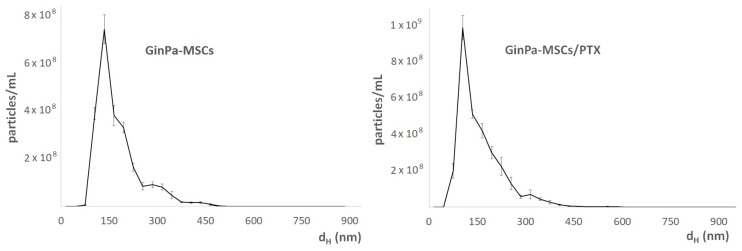
Size distribution analysis of the secretomes of GinPaMSCs and GinPaMSCs/PTX, analyzed by nanoparticle tracking assay (NTA). In both samples, several populations of vesicles were present at 200−300 nm.

NTA analysis of the secretome evidenced the presence of different populations of vesicles and allowed us to clarify their number distribution in the samples. In particular, the whole secretome showed three different populations at about 135 nm, 200–300 nm, and 435 nm (Figure 5). The latter was not detected in the samples obtained by GinPaMSCs/PTX, which in general showed a narrowed particle size distribution, as exemplified in Figure 5. GinPaMSCs/PTX released a higher number of extracellular vesicles than untreated GinPaMSCs (3.01 × 10^9^ ± 1.19 × 10^8^ versus 2.37 × 10^9^ ± 6.80 × 10^7^ particles/mL, respectively) but the difference was not statistically relevant.

### 3.5. Paclitaxel Dosage in EV Fractions of GinPaMSCs and GinPaMSCs/PTX Secretomes

**Figure 6:**
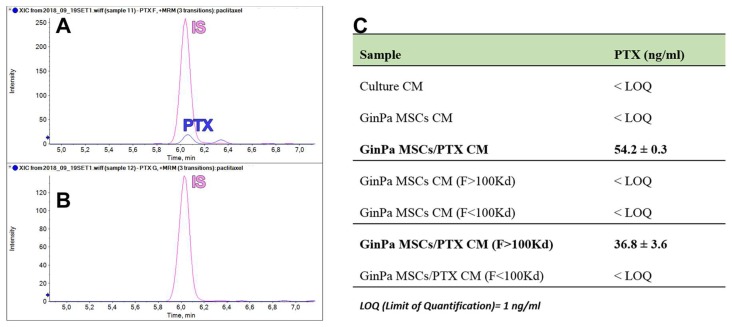
PTX dosage by mass spectrometry in EV fractions. The chromatogram identification of PTX peaks by LC–MS/MS retention time: (**A**) F > 100 kDa fraction and (**B**) F < 100 kDa fraction, in which no PTX peaks were appreciable. PTX was quantified successfully in both the unfractioned secretome and the F > 100 kDa fraction of PTX-treated GinPaMSCs (**C**).

The validated LC–MS/MS method was successfully applied to quantify PTX in the different samples of conditioned media from GinPaMSCs and GinPaMSCs/PTX: Unfractionated secretomes and ultra-filtered fractions (F > 100 kDa and F < 100kDa). PTX was present in both the whole secretome and the F > 100kDa fraction of PTX-treated GinPaMSCs, suggesting the incorporation or the unspecific bind of PTX to EVs (Figure 6A). No signal related to PTX was detected in the secretome of untreated GinPaMSCs used as negative controls (data not shown) or in ultra-filtered <100 GinPaMSCs/PTX samples (Figure 6C).

### 3.6. Anticancer Activity of EVs from GinPaMSCs and GinPaMSCs/PTX Secretomes 

**Figure 7:**
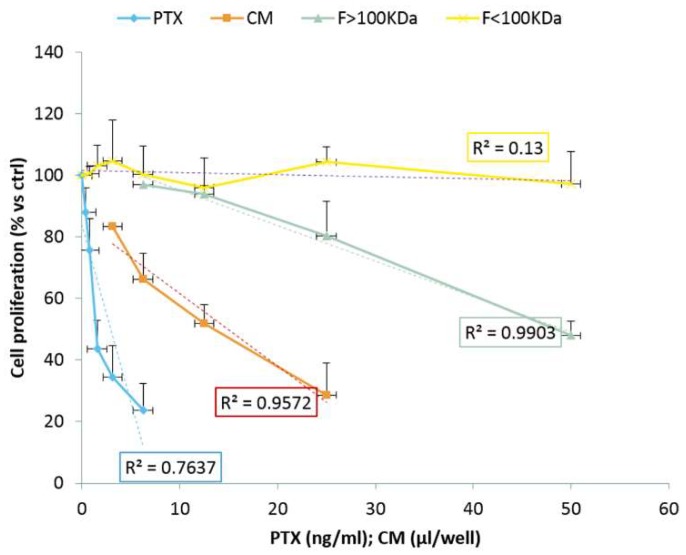
The anticancer activity of EVs from GinPaMSCs and GinPaMSCs/PTX tested against squamous cancer cells (SCC154). The activity is expressed as percentage of cell growth, normalized on the effect of EVs secreted by untreated GinPaMSCs used as controls (OD: 1.28 ± 0.09 was considered 100% proliferation). Data is reported as the mean ± standard deviation of three independent experiments.

The anticancer activity of EVs secreted by GinPaMSCs/PTX was tested against squamous cancer cells (SCC154) (Figure 7). The activity is expressed as percentage of cell growth, normalized on the effect of EVs secreted by untreated GinPaMSCs used as controls. The fraction F < 100 kDa did not exert any activity against the cancer cell proliferation, with a non-significant coefficient of correlation (*R*^2^ = 0.13). The fraction F > 100 KDa produced a significant dose-dependent inhibition of cancer cell growth, also confirmed by regression analysis (*R*^2^ = 0.99), that is similar to the inhibition produced by the unfractioned secretome (CM) (*R*^2^ = 0.95). These results agree with the mass spectrometry analysis that demonstrated the presence of PTX in both CM and in the F > 100 kDa (Figure 6).

## 4. Discussion

Our data confirm that MSCs from gingival papilla (GinPaMSCs) have a significant resistance to PTX that tested until 10,000 ng/mL, reducing the cell viability by about 20% and blocking cells in the G2/M cycle phase (Figure 1). This evidence agrees with literature data which demonstrated that the treatment of MSCs with PTX reduces their proliferation activity, migration ability, and some differentiation potentials, without significantly affecting their viability [32,33]. Of course, the analyses of secretomes of GinPaMSCs identified the presence of cytokines that did not modulate, stimulate, or inhibit the growth of four different tumor cell lines (Figure 2A). On the contrary, the secretome from the cells primed with PTX exerted a dramatic dose–response inhibition on both CFPAC-1 (pancreatic carcinoma cells) and SCC-154 (squamous carcinoma cells) (Figure 2B,C). After priming of GinPaMSCs by PTX, a little modulation of cytokine production was observed that may be considered ineffective on cancer cell growth, suggesting that the dramatic anticancer activity exerted by GinPaMSC/PTX secretomes can be mainly due to the presence of the drug secreted by PTX-primed GinPaMSCs. This result is also confirmed by the significant toxicity of GinPaMSCs/PTX only against CFPAC-1 and SCC-154, whereas cancer cells co-cultured in the presence of unprimed GinPaMSCs showed no sign of toxicity (Figure 3). Based on our previous experience with an established murine MSC line (SR4987) [20], we here investigated if primary human MSCs, particularly the gingival-derived MSCs, were able to process and then deliver PTX associated to exosomes or microvesicles. TEM analysis of GinPaMSCs secretomes demonstrated the presence of microvesicles at different sizes (Figure 4). This result was further confirmed both by dynamic light scattering (DLS) and nanoparticle tracking assay (NTA). In particular, DLS indicated the presence of a population of microvesicles ranging between 200 and 300 nm both in the secretome of GinPaMSCs and that of GinPaMSCs/PTX. Moreover, it can be assumed that isolated microvesicles shared the same origin, since the same amount of phospholipids was detected in both types of cells (Table 1). The study on size distribution by NTA evidenced three different populations in the GinPaMSCs secretome, whereas the population at about 435 nm was not detected in the Gin-PaMSCs/PTX secretome (Figure 5). This small discrepancy between NTA and DLS data depends only on the scattering intensity, because the larger the particle, the higher the contribution to the total signal. Instead, NTA allows us to provide a more reliable distribution in number. In any case, besides the slight differences in particle size distributions, it is important to highlight that the GinPaMSCs treated with PTX released a slightly higher number of EVs with respect to untreated GinPaMSCs. This difference in not statistically relevant, but this trend confirmed that PTX treatment did not affect the microvesicle biogenesis of GinPaMSCs. 

The anti-proliferation activity of EVs was studied in both the unfractioned secretome (i.e., CM) and its fractions. The results confirmed that the anticancer activity was due only to the fraction containing MVs (F > 100KDa). As expected, no activity was found in the unfractioned secretomes of untreated MSCs (Figure 7), while it was a little surprising that the F < 100KDa fraction from GinPaMSCs/PTX did not contain PTX and did not present activity. This apparent discrepancy can be explained considering that a lipophilic drug, such as PTX, can be easily adsorbed and retained by filters. However, the present research was designed as a qualitative study with the main purpose to demonstrate the ability of GinPaMSCs to secrete PTX associated with EVs. A deep investigation is currently in progress to optimize the incorporation and/or release of PTX in the attempt to produce large batches of EVs by using bioreactors.

The microvesicles secreted by GinPaMSCs/PTX were active against two tumor cell lines, namely pancreatic carcinoma (CFPAC1) and tongue squamous cell carcinoma (SCC154) cell lines. As found in the preliminary screening (Figure 1), CM from GinPaMSCs/PTX was active on different tumor cell lines, and we focused the study on a human SCC model that could express an important anatomical/histological homology with the origin of MSCs generated by gingival tissue. In general, MSCs are considered as an important source of EVs and/or exosomes, and are under investigation for their role both in tumor progression and in drug delivery for tumor therapy and regenerative medicine [34,35,36,37]. As reported [20,38], upon in vitro exposure to high concentrations of PTX, MSCs can “load” the drug and deliver it by means of EVs so that the PTX-loaded EVs acquire strong anti-tumor effects on human cancers both in vitro and in vivo. Even if cancer cell-derived exosomes were proposed as effective carriers of PTX to their parental cells [39], our results demonstrated for the first time that human gingival MSCs produce a significant amount of EVs and can be considered an ideal candidate for the large production of EVs and/or exosomes. If primed with PTX, these cells can also release the drug associated to EVs and/or exosomes acting as “natural anticancer liposomes”. Among the different MSC sources, our study suggests that gingival MSCs, which present an important homology with tumors originated from oral tissues (such as SCC), could be obtained with a minimally invasive procedure and easily expanded.

Currently, of paramount importance are preclinical studies, which can further corroborate the advantages of EVs. As a matter of fact, EVs could be easily manufactured in large batches by reducing the cost due to the need to personalize the cellular products, and have a superior safety profile in therapy (e.g., reduced risk related to ectopic tissue formation, microvasculature infusion toxicity, rejection) with respect to MSCs. Furthermore, the use of EVs in therapy gives the possibility to manage high drug concentrations in a minimal volume, improving the storage, the transport, and the infusion procedures [40,41].

## Figures and Tables

**Table 1:** Main physico-chemical features of the secretome.

Samples		D_H_ (nm)	ζ-potential (mV)	Phospholipids (mM)
GinPaMSCs	unfractionated	242 ± 34	−16.6 ± 0.2	0.32 ± 0.06
	Ultra-filtrated (F > 100 kDa)	430 ± 33	−20.9 ± 0.3	0.49 ± 0.07
GinPaMSCs/PTX	unfractionated	303 ± 23	−18.1 ± 2.3	0.33 ± 0.09
	Ultra-filtrated (F > 100 kDa)	385 ± 19	−22.7 ± 0.3	0.62 ± 0.15
